# A formylpeptide receptor, FPRL1, acts as an efficient coreceptor for primary isolates of human immunodeficiency virus

**DOI:** 10.1186/1742-4690-5-52

**Published:** 2008-06-25

**Authors:** Nobuaki Shimizu, Atsushi Tanaka, Takahisa Mori, Takahiro Ohtsuki, Aliful Hoque, Atsushi Jinno-Oue, Chatchawann Apichartpiyakul, Shigeru Kusagawa, Yutaka Takebe, Hiroo Hoshino

**Affiliations:** 1 Department of Virology and Preventive Medicine, Gunma University Graduate School of Medicine, Showa-machi, Maebashi, Gunma 371-8511, Japan; 2 21st Century COE Program, Gunma University Graduate School of Medicine, Showa-machi, Maebashi, Gunma 371-8511, Japan; 3 Department of Microbiology, Faculty of Medicine, Chiang Mai University, Chiang Mai 50200, Thailand; 4 Laboratory of Molecular Virology and Epidemiology, AIDS Research Center, National Institute of Infectious Diseases, Toyama, Shinjuku-ku, Tokyo 162-8640, Japan

## Abstract

**Background:**

More than 10 members of seven-transmembrane G protein-coupled receptors (GPCRs) have been shown to work as coreceptors for human immunodeficiency virus type 1 (HIV-1), HIV type 2 (HIV-2), and simian immunodeficiency viruses (SIVs). As a common feature of HIV/SIV coreceptors, tyrosine residues are present with asparagines, aspartic acids or glutamic acids in the amino-terminal extracellular regions (NTRs).

We noticed that a receptor for N-formylpeptides, FPRL1, also contains two tyrosine residues accompanied by glutamic acids in its NTR. It was reported that monocytes expressing CCR5 and FPRL1 in addition to CD4 are activated by treatment with ligands or agonists of FPRL1. Activated monocytes down-modulate CCR5 and become resistant to infection by HIV-1 strains. Thus, FPRL1 plays important roles in protection of monocyptes against HIV-1 infection. However, its own coreceptor activity has not been elucidated yet. In this study, we examined coreceptor activities of FPRL1 for HIV/SIV strains including primary HIV-1 isolates.

**Results:**

A CD4-transduced human glioma cell line, NP-2/CD4, is strictly resistant to HIV/SIV infection. We have reported that when NP-2/CD4 cells are transduced with a GPCR having coreceptor activity, the cells become susceptible to HIV/SIV strains. When NP-2/CD4 cells were transduced with FPRL1, the resultant NP-2/CD4/FPRL1 cells became markedly susceptible to some laboratory-adapted HIV/SIV strains. We found that FPRL1 is also efficiently used as a coreceptor by primary HIV-1 isolates as well as CCR5 or CXCR4.

Amino acid sequences linked to the FPRL1 use could not be detected in the V3 loop of the HIV-1 Env protein. Coreceptor activities of FPRL1 were partially blocked by the forymyl-Met-Leu-Phe (fMLF) peptide.

**Conclusion:**

We conclude that FPRL1 is a novel and efficient coreceptor for HIV/SIV strains. FPRL1 works as a bifunctional factor in HIV-1 infection. Namely, the role of FPRL1 in HIV-1 infection is protective and/or promotive in different conditions. FPRL1 has been reported to be abundantly expressed in the lung, spleen, testis, and neutrophils. We detected mRNA expression of FPRL1 in 293T (embryonal kidney cell line), C8166 (T cell line), HOS (osteosarcoma cell line), Molt4#8 (T cell line), U251MG (astrocytoma cell line), U87/CD4 (CD4-transduced glioma cell line), and peripheral blood lymphocytes. Roles of FPRL1 in HIV-1 infection *in vivo *should be further investigated.

## Background

More than 10 members of seven-transmembrane G protein-coupled receptors (GPCRs) support the entry of human immunodeficiency virus type 1 (HIV-1), HIV type 2 (HIV-2), and simian immunodeficiency viruses (SIVs) into target cells as coreceptors in collaboration with the primary receptor CD4 [1,2].

A chemokine receptor (CKR), CXCR4, was firstly shown to work as a coreceptor for HIV-1 strains [3]. Next, a CKR, CCR5, was also identified as a coreceptor for HIV-1 strains [4,5]. Infection of T cells or macrophages with HIV-1 strains that can use CCR5 as a coreceptor (i. e., R5 strains) is an initial event in the establishment of HIV-1 infection *in vivo*, since peoples harboring mutant alleles in the CCR5 gene have been found to be highly resistant to infection by HIV-1, even if they have been estimated to have repeatedly been exposed to the viruses [6,7]. During progression of stages in HIV-1 infection, HIV-1 strains that use CXCR4 as a coreceptor (X4 strains), especially subtype B strains, have been detected [8]. HIV-1 strains that can use both CCR5 and CXCR4 (R5-X4 strains) often emerge, but their roles in pathogenesis of acquired immune deficiency syndrome (AIDS) remain to be elucidated [9]. Thus, these two coreceptors, CCR5 and CXCR4, have been thought to play major roles in HIV-1 infection and the development of related disorders.

Some GPCRs, especially CKRs, play a major role in migration of lymphocytes (chemotaxis) and consequently in the development of inflammation together with their ligands, chemotactic cytokines (chemokines) [10-12]. CKRs are classified into five groups, CC-, CXC-, CX3C-, and XC-CKRs, and other CKRs according to the well conserved amino acid motifs of their ligands [13]. Some CKRs have been shown to act as coreceptors for HIV-1, HIV-2 or SIV (HIV/SIV) strains. In addition to CCR5 and CXCR4, three CC-CKRs, CCR2b, CCR3, CCR8, and D6 have been shown to be alternative coreceptors mainly used by R5 or X4 HIV-1 strains [14-16]. Two CXC-CKRs, CXCR5/BLR1 and CXCR6/BONZO, act as coreceptors for several HIV/SIV strains [17-19]. A CX3C-CKR, CX3CR1/V28, functions as a coreceptor for several HIV-1 strains [20]. In addition to CKRs, several GPCRs, e. g., APJ [21], ChemR23 [22], GPR1 [23], GPR15 [24], RDC1 [25], and the leukotrien B4 receptor LTB4 [26], have been reported to work as coreceptors for HIV/SIV strains. However, the roles of these coreceptors in HIV-1 infection *in vivo *have not been elucidated [27].

We noticed that as a common feature of most CKRs, as well as HIV/SIV coreceptors, tyrosine residues with asparagines, aspartic acids or glutamic acids are present in the amino-terminal extracellular regions (NTRs) [28,29]. A receptor for N-formylpeptides, FPRL1, also contains two tyrosine residues accompanied by glutamic acids in its NTR [30]. FPRL1 has been reported to be expressed in the lung, spleen, and testis, and in neutrophils, and to play an important role in the activation of neutrophils [31]. Monocytes expressing FPRL1 in addition to CD4 are activated by treatment with ligands or agonists of FPRL1. Activated monocytes down-modulate CCR5 and thus become resistant to infection by R5 HIV-1 strains. [32]. In this report, we demonstrate that FPRL1 itself has the capacity to support the entry of various HIV/SIV strains, including primary HIV-1 isolates, into target cells as a novel coreceptor.

## Results

### FPRL1 as a novel candidate HIV/SIV coreceptor

Major HIV/SIV coreceptors, CCR5 and CXCR4, contain tyrosines and these tyrosines in NTRs of CCR5 and CXCR4 have been demonstrated to be necessary for their coreceptor activities [28,33]. All CKRs reported to have HIV/SIV coreceptor activities harbor tyrosines in their NTRs. Most non-CKR GPCRs that were reported to function as HIV/SIV coreceptors also harbor tyrosines accompanied by aspartic acids, glutamic acids or asparagines in their NTRs (Additional file 1).

**Figure 1:**
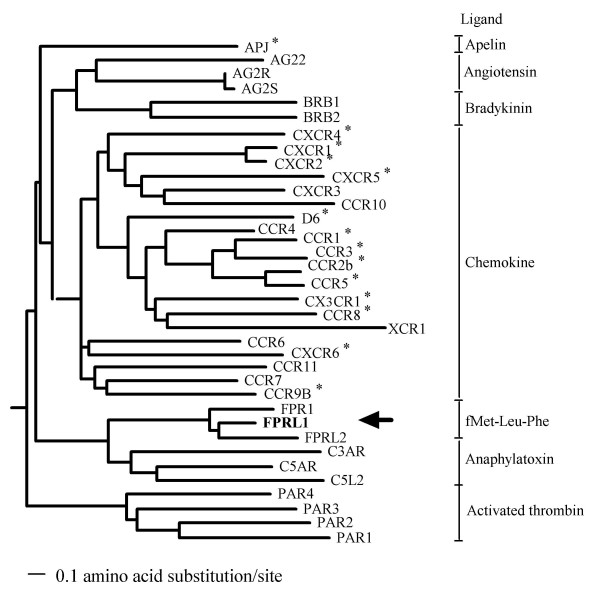
**Phylogenetic tree of peptide receptors belonging to the GPCR family**. The phylogenetic tree for 20 CKRs and 16 GPCRs related to CKRs was constructed by the ClustalW program [72] according to the methods described in the DDBJ website (National Institute of Genetics, Center for Information Biology and DNA Databank of Japan, ). FPRL1 is indicated by the arrow. GPCRs reported to function as HIV/SIV coreceptors are indicated by "*".

Therefore, to discover a novel candidate coreceptors of HIV/SIV, we constructed a phylogenetic tree of peptide receptors for 36 GPCRs containing reported HIV/SIV coreceptors (20 CKRs, and 16 non-CKR GPCRs) using the ClustalW program [72] (Fig. 1). The peptide receptors were clustered into several distinct branches corresponding to the subfamilies of GPCRs. In this phylogenetic tree, we found that CKRs were closely related to each other and that all of the formylpeptide receptors reported so far, FPRL1, FPRL2 and FPR1, constituted a distinct subgroup, closely located at positions near CKRs and anaphylatoxin receptors, some of which have been demonstrated to act as coreceptors for HIV/SIV [22]. FPRL1, unlike FPRL2 or FPR1, has tyrosine residues accompanied by asparagines, aspartic acids, and glutamic acids in its NTR (see Additional file 1). Therefore, we focused on a formylpeptide receptor, FPRL1, as a novel candidate coreceptor for HIV/SIV.

### Susceptibility of NP-2/CD4/FPRL1 cells to cell line-adapted HIV-1 strains

**Figure 2:**
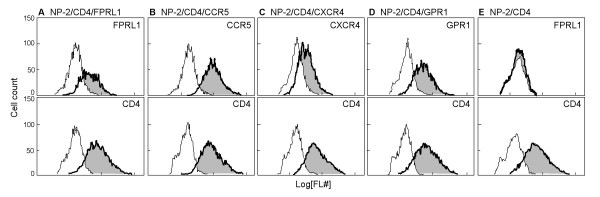
**Expression of GPCR mRNA in various types of human cells detected by RT-PCR**. (A) Relative amount of mRNA expression for CCR5, CXCR4, FPRL1 and GPR1 in NP-2/CD4 cells expressing the coreceptors. RT-PCR was done using serially diluted (1:1, 1:10, 1:100, 1:1000, and 1:10000) cDNA reverse-transcribed from the total RNA. As a control, the expression level of GAPDH mRNA in each cDNA preparation was determined by RT-PCR. (B) mRNA expression for four GPCRs in 11 kinds of human cells as detected by RT-PCR using the specific primers. As a control, the expression level of GAPDH mRNA in each cDNA preparation was determined by RT-PCR. The PCR primers amplify 1,377 (CD4), 1,059 (CCR5 and CXCR4), 1,056 (FPRL1), 1,068 (GPR1), and 1,008 (GAPDH) base-pair DNA fragments when these genes are expressed in the cells. Expression level, (-~++) were determined by intensities of amplified DNA bands compared to those of the corresponding controls (GAPDH).

FPRL1 ORF DNA was amplified using cDNA made from C8166 cell mRNA and cloned into the expression vector pCX-bsr. The expression level of the FPRL1 gene in NP-2/CD4 cells transfected with it, NP-2/CD4/FPRL1, was determined by RT-PCR. mRNA expressions of CCR5, CXCR4, and GPR1 were also detected in NP-2/CD4/CCR5, NP-2/CD4/CXCR4, and NP-2/CD4/GPR1 cells by RT-PCR, respectively. A comparison of the intensity of each PCR band shown in Fig. 2A suggests that the amount of FPRL1 mRNA in NP-2/CD4/FPRL1 cells was 10–100 fold more abundant than the mRNA of CCR5 in NP-2/CD4/CCR5 cells, CXCR4 in NP-2/CD4/CXCR4 cells or GPR1 in NP-2/CD4/GPR1 cells.

**Figure 3:**
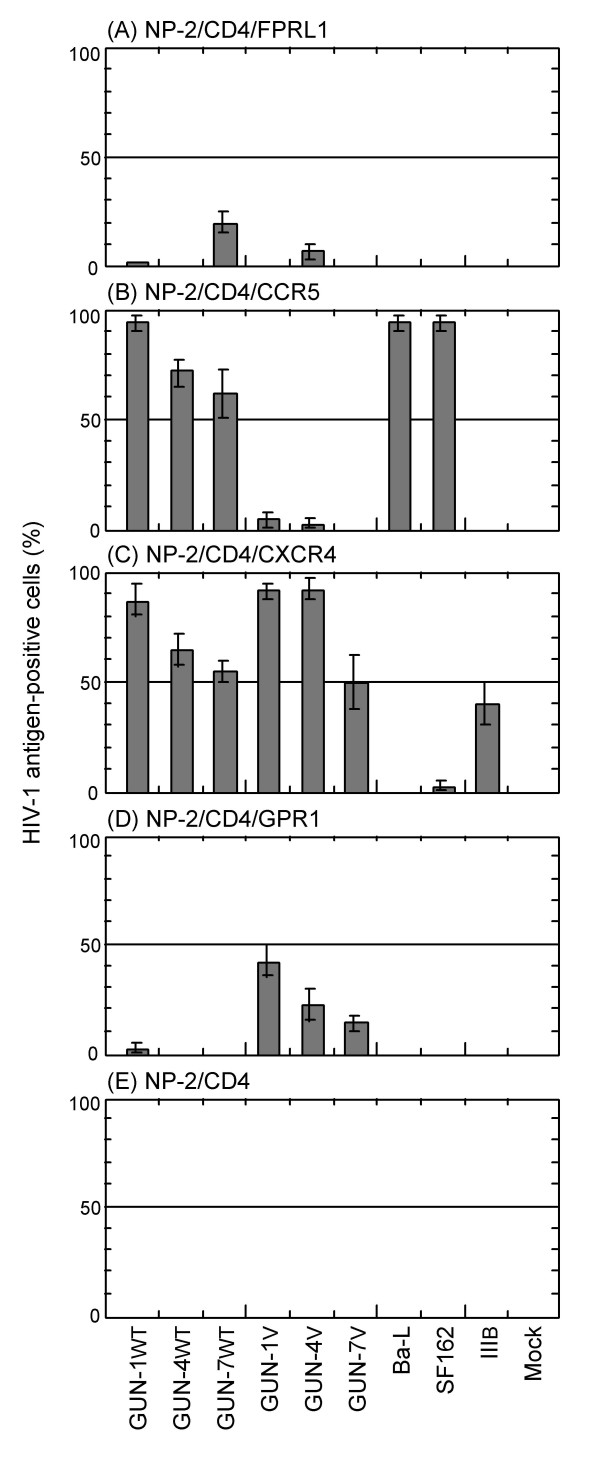
**Use of FPRL1, CCR5, CXCR4 or GPR1 as a coreceptor by various cell line-adapted HIV-1 strains**. Cells were inoculated with nine HIV-1 strains. The susceptibilities of the cells were determined by IFA six days after viral inoculation. The coreceptor uses of these HIV-1 strains are summarized (see Additional file 2). NP-2/CD4 cells were also tested up to eight days after inoculation and were completely resistant to all the HIV-1 strains examined.

To clarify whether FPRL1 has the ability to serve as a coreceptor, the susceptibility of NP-2/CD4/FPRL1 cells to nine cell line-adapted HIV-1 strains was investigated. NP-2/CD4/FPRL1 cells were found to be susceptible to the GUN-1WT, GUN-4V, and GUN-7WT cell-line-adapted HIV-1 strains: approximately 0.5, 5 and 30% of the cells became HIV-1 antigen-positive on day 6 after infection, respectively (Fig. 3A). NP-2/CD4/FPRL1 cells were resistant to infection by IIIB, Ba-L, GUN-1V, GUN-4WT, GUN-7V, and SF162 strains: less than 0.1% cells were HIV-1 antigen-positive on day 6 after infection. NP-2/CD4 cells, in which no expression of the FPRL1, CCR5, CXCR4, or GPR1 gene was detected by RT-PCR (Fig. 2B), were completely resistant to infection by all HIV-1 strains tested (Fig. 3E), as previously described [49]. Thus, FPRL1 enabled infection of several cell line-adapted HIV-1 strains as a coreceptor.

As controls, the susceptibilities of NP-2/CD4/CXCR4, NP-2/CD4/CCR5, and NP-2/CD4/GPR1 cells to HIV-1 strains were also examined. NP-2/CD4/CXCR4 cells were highly susceptible to all HIV-1 strains, except the Ba-L and SF162 strains, when tested on day 6 after infection (Fig. 3B), while NP-2/CD4/CCR5 cells were highly susceptible to five HIV-1 strains, Ba-L, GUN-1WT, GUN-4WT, GUN-7WT, and SF162, but not to the IIIB strain (Fig. 3C). NP-2/CD4/GPR1 cells were susceptible to three HIV-1 variants, GUN-1V, GUN-4V, and GUN-7V, but not to three HIV-1 strains, IIIB, Ba-L, and SF162 (Fig. 3D). The coreceptor uses of the cell line-adapted HIV-1 strains are summarized (see Additional file 2) and as follows: IIIB (coreceptor use, X4), Ba-L (R5), GUN-1WT (FPRL1-R5-X4), GUN-1V (GPR1-X4), GUN-4WT (R5-X4), GUN-4V (FPRL1-GPR1-X4), GUN-7WT (FPRL1-R5-X4), GUN-7V (GPR1-X4), and SF162 (R5). We have reported that there are one or two amino acid mutations in the V3 region of gp120 between GUN-1WT and GUN-1V, between GUN-4WT and GUN-4V, and between GUN-7WT and GUN-7V [59]. Our results suggest that amino acid sequences of the V3 region markedly affected FPRL1 use as a coreceptor by HIV-1 strains.

### FPRL1 as a coreceptor for primary isolates of HIV-1

Next, we investigated whether FPRL1 also acts as a coreceptor for primary HIV-1 isolates. HIV-1 strains, AG204, AG206, AG208, HCM303, HCM305, HCM308, HCM309, HCM342, mIDU101, and mSTD104, were isolated from PBLs derived from HIV-1-infected Vietnamese or Myanmanese subjects and had been propagated only in PBLs before this experiment.

**Figure 4:**
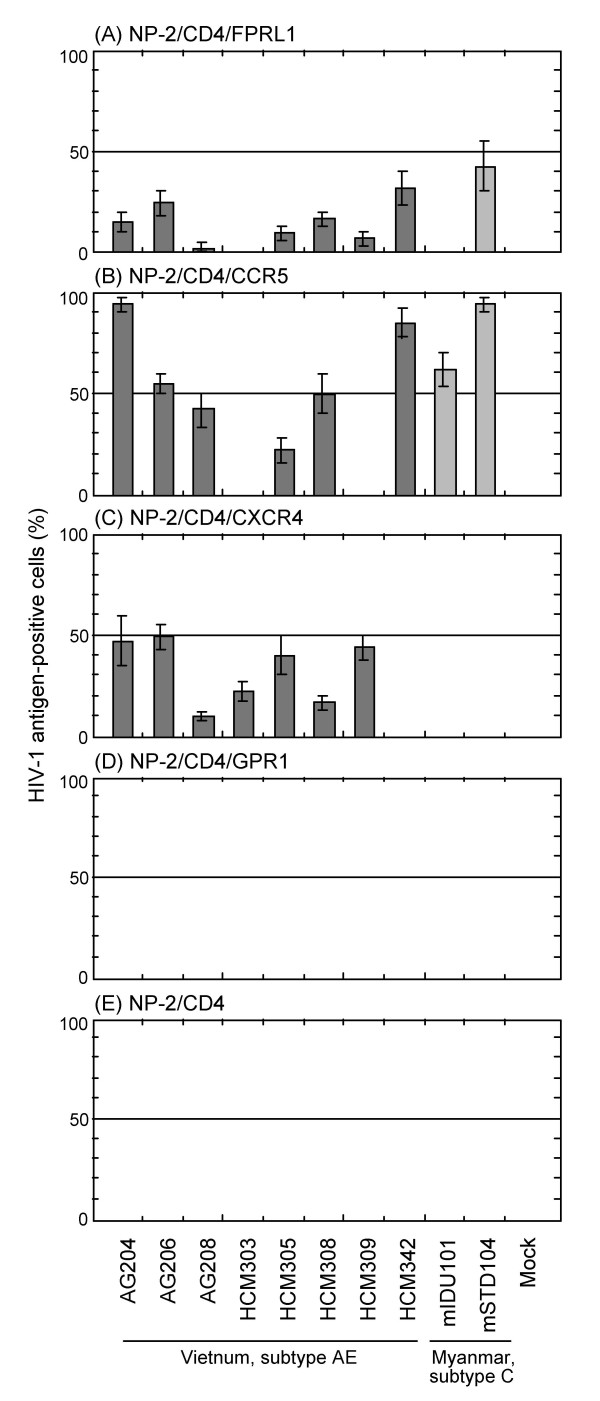
**Use of FPRL1, CCR5, CXCR4 or GPR1 as a coreceptor by various primary HIV-1 isolates**. The susceptibilities of cells to HIV-1 isolates were determined by IFA six days after viral inoculation. NP-2/CD4 cells were completely resistant to all these HIV-1 isolates (E). The origins and subtypes of these primary isolates are summarized (see Additional file 2).

**Figure 5:**
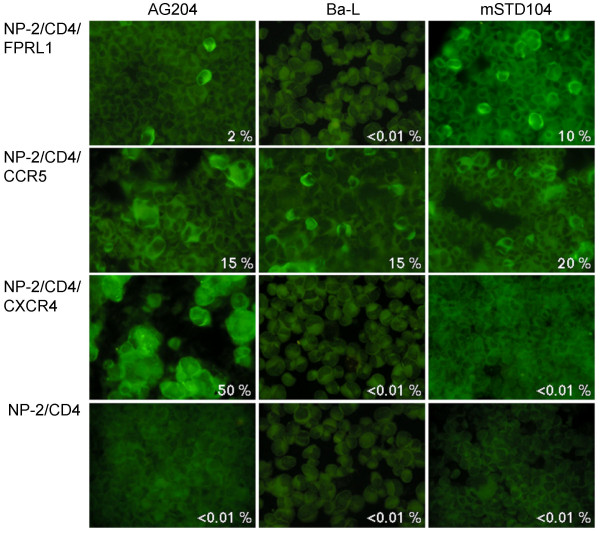
**The susceptibilities of NP-2/CD4/GPCR cells to HIV-1 strains**. Cells were infected with two primary HIV-1 isolates, AG204 and mSTD104, and a cell line-adapted strain, Ba-L. Six days after infection, cells positive for HIV-1 antigens were detected by IFA using a fluorescence microscope. Percentage of cells judged to be positive for IFA are shown.

When NP-2/CD4/FPRL1 cells were exposed to these isolates, the cells were found to be clearly susceptible to AG204, AG206, HCM308, HCM342, and mSTD104 isolates: 20, 30, 15, 40, and 60% cells, respectively, became HIV-1 antigen-positive by IFA on day 6 after infection and syncytia were formed (Figs. 4A and 5). A large number of syncytia were formed in the infection of NP-2/CD4/FPRL1 cells with the AG206, HCM342, and mSTD104 isolates, suggesting that replication of HIV-1 efficiently occurred in these cells (data not shown). NP-2/CD4/FPRL1 cells also demonstrated lower, but clear susceptibilities to other isolates, AG208, HCM305, and HCM309. NP-2/CD4/FPRL1 cells were not susceptible to HCM303 and mIDU101 isolates. Thus, eight out of the ten primary HIV-1 isolates could infect NP-2/CD4/FPRL1 cells.

NP-2/CD4/CCR5 cells were highly susceptible to AG204, AG206, HCM308, HCM342, mIDU101, and mSTD104 isolates (Fig. 4B), and slightly susceptible to AG208, HCM303, HCM305, and HCM309 isolates.

NP-2/CD4/CXCR4 cells showed a high susceptibility to AG204, AG206, AG208, HCM303, HC305, HCM308, and HCM309 isolate (Fig. 4C), while less than 1% of HIV-1 antigen-positive cells were detected after infection with HCM342, mIDU101, or mSTD104 isolates. NP-2/CD4/GPR1 and NP-2/CD4 cells were resistant to infection by all of these primary isolates (Figs. 4D, 4E, and 5).

The phenotypes of the coreceptor uses of the primary HIV-1 isolates were as follows (see Additional file 2): FPRL1-R5-X4 use, AG204, AG206, AG208, HCM305, and HCM308; FPRL1-R5 use, HCM342 and mSTD104; FPRL1-X4 use, HCM309; R5 use, mIDU101, and X4 use, HCM303. Thus, FPRL1 can work as a coreceptor not only for cell line-adapted HIV-1 strains but also for primary HIV-1 isolates.

### Amino acid sequences of the V3 domain of the HIV/SIV Env protein

Cell line-adapted HIV-1 strains, GUN-4V (GPR1-X4) and GUN-7WT (R5-X4), could not use FPRL1 as a coreceptor, whereas their related strains, GUN-4WT and GUN-7V, with one or two amino acid substitutions in the V3 region, could use FPRL1 (Fig. 3 and see Additional file 2). This finding raised the possibility that a determinant of the FPRL1 use of HIV-1 as a coreceptor lies in the V3 region. Therefore, we determined the amino acid sequences of the V3 regions of primary HIV-1 strains propagated in NP-2/CD4 cells expressing one of the coreceptors. DNA regions coding for the V3 domain of gp120 were amplified by PCR using cellular DNA of NP-2/CD4/FPRL1 cells infected with AG204, AG206, HCM305, HCM309, HCM342, or mSTD104 strains and NP-2/CD4/CXCR4 cells infected with the HCM303 strain as templates. These amplified DNAs were cloned into the TA-cloning vector pTarget and their nucleotide sequences were determined.

When their nucleotide sequences were compared with those of HIV-1 isolates submitted to the Genbank and reported previously [34], several nucleotide substitutions were observed in the V3 sequences of the primary isolates infecting NP-2/CD4/CCR5, NP-2/CD4/CXCR4, or NP-2/CD4/FPRL1 cells (data not shown).

No amino acid substitution, however, was detected in the V3 domains, because all the nucleotide substitutions detected in the V3 domains were synonymous (see Additional file 3). Single-amino acid substitutions were detected in the C3 domain of AG206, HCM303, and HCM342 strains propagated in FPRL1-expressing cells. These findings indicate that subtype C or AE HIV-1 strains propagated in PBLs and those propagated in NP-2/CD4 cells expressing CCR5, CXCR4 or FPRL1 have the identical amino acid sequences in the V3 domains. Nevertheless, five HIV-1 strains using FPRL1, GUN-1WT, GUN-7WT, HCM305, HCM309, and HCM342, had threonine at the 13th amino acid position of the V3 region, while two HIV-1 strains which did not use FPRL1 as a coreceptor, GUN-4WT and HCM303, had serine at this position. The amino acids at this position may be responsible for determining FPRL1 use by these HIV-1 strains.

HIV-1 samples produced by NP-2/CD4/FPRL1 cells that had been infected with the AG204, AG206, HCM308, and HCM342 strains could use CCR5 as a coreceptor (data not shown). These results suggest that the primary HIV-1 samples are not a mixture of FPRL1-tropic virus and R5-tropic virus, and that HIV-1 isolates using FPRL1 can also use CCR5 as a coreceptor.

### FPRL1 as a coreceptor for HIV-2 and SIV strains

**Figure 6:**
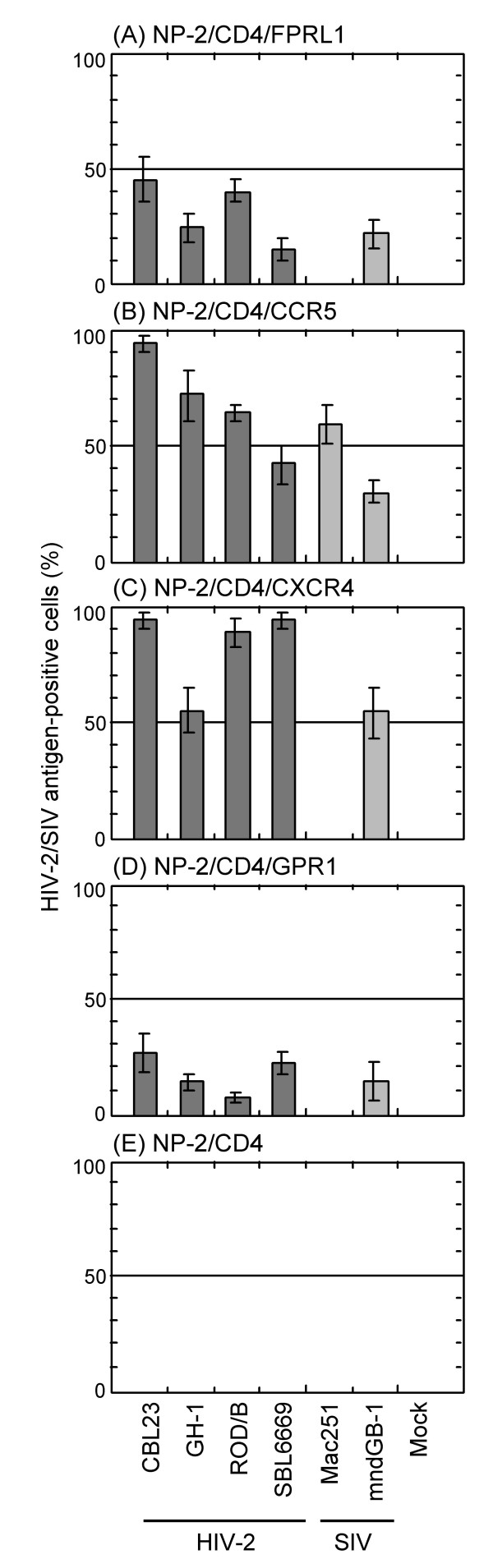
**Use of FPRL1, CCR5, CXCR4 or GPR1 as a coreceptor by HIV-2 and SIV strains**. The susceptibilities of cells to four HIV-2 strains and two SIV strains were determined by IFA six days after viral inoculation. NP-2/CD4 cells were completely resistant to these HIV-2 strains (E). These results are summarized (see Additional file 2).

Next, we tested a coreceptor activity of FPRL1 for four HIV-2 and SIV strains. NP-2/CD4/FPRL1 cells were highly susceptible to two HIV-2 strains CBL23 and ROD/B: about 60% of cells became HIV-2 antigen-positive on day 6 after infection (Fig. 6A). As for the GH-1 and SBL6669 HIV-2 strains, and mndGB-1 SIV strain, 30, 15, and 30% of the cells, respectively, were infected. NP-2/CD4/FPRL1 cells were, however, resistant to the R5 SIV strain mac251.

As reported previously [49], NP-2/CD4/CCR5 cells were susceptible to four HIV-2 strains (CBL23, GH-1, ROD/B, and SBL6669) and two SIV strains (mac251 and mndGB-1) (Fig. 6B). NP-2/CD4/CXCR4 and NP-2/CD4/GPR1 cells were susceptible to these four HIV-2 strains and the mndGB-1 strain (Figs. 6C and 6D), but not to the mac251 strain. Thus, the coreceptor uses of HIV-2 and SIV strains are summarized (see Additional file 2). FPRL1 may work as a coreceptor for HIV-2 and SIV strains with an R5-X4-GPR1 phenotype, but not those with an R5 phenotype.

### CD4 dependency of the coreceptor activity of FPRL1

**Figure 7:**
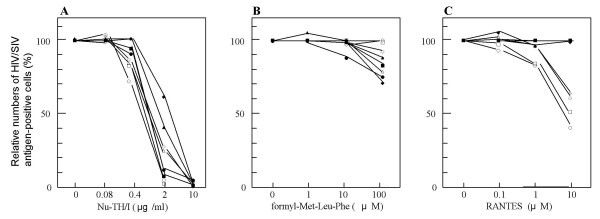
**Effects of anti-CD4 MoAb, the fMLF peptide, and RANTES on the susceptibility of cells to HIV/SIV strains**. NP-2/CD4/CCR5 (open symbols) and NP-2/CD4/FPRL1 (closed symbols) cells were pretreated with anti-CD4 MoAb Nu-TH/I (A), the fMLF peptide (B) or RANTES for two hours at 37°C (C), and then inoculated with two HIV-1 strains, GUN-7WT (○ and ●) and HCM342 (□ and ■), HIV-2 CBL23 strain (△ and ▲), and SIV mndGB-1 strain (◇ and ◆). Six days after infection, cells positive for HIV-1 antigens were detected by IFA using a fluorescence microscope.

Some HIV-2 strains have been shown to enter CD4-negative cells, and this entry is mediated through coreceptors [35,36]. To clarify whether infection of HIV/SIV mediated through FPRL1 is dependent on CD4 or not, NP-2/CD4/FPRL1 and NP-2/CD4/CCR5 cells were pretreated with serially-diluted anti-CD4 MoAb, NuTH/I, before inoculation. Relative percentages of infected cells are shown in Fig. 7A. NuTH/I MoAb (10 μg/ml) almost completely inhibited infection of NP-2/CD4/FPRL1 cells, as well as NP-2/CD4/CCR5 cells, with all HIV/SIV strains tested, *i. e.*, GUN-7WT, HCM342, CBL23, and mndGB-1, suggesting that FPRL1 mediates infection of HIV/SIV as a coreceptor, *i. e*., in a CD4-dependent manner.

### Partial inhibition of the coreceptor activity of FPRL1 by the fMLF peptide

It has been reported that the coreceptor functions of CCR5 and CXCR4 for HIV-1 infection can be inhibited by their ligands, RANTES and SDF-1β, respectively [37,38]. Some ligands have been used as starting materials to find and develop anti-HIV reagents. We examined the inhibitory effects of an FPRL1 ligand, fMLF peptide, on infection with HIV/SIV.

NP-2/CD4/FPRL1 cells were pretreated with the fMLF peptide (100 μg/ml). As a control, NP-2/CD4/CCR5 cell were also pretreated with a chemokine, RANTES, a ligand for CCR5. As shown in Fig. 7B, the fMLF peptide (100 μg/) showed a partial inhibitory effect on infection of NP-2/CD4/FPRL1 cells with GUN-7WT, HCM342, CBL23, or the mndGB-1 strain. Infection of NP-2/CD4/CCR5 cells with CBL23 and mndGB-1, but not with the GUN-7WT or HCM342 strains, was also partially blocked by the peptide, suggesting that the fMLF peptide may have inhibitory effects on infection of CCR5-positive cells by several strains of HIV/SIV. The difference in inhibitory effects of the fMLF peptide may reflect the HIV/SIV strain-dependent interaction with CCR5. On the contrary, as shown in Fig. 7C, a chemokine, RATES, had hardly any effect on infection of NP-2/CD4/FPRL1 cells with the HIV/SIV strain. RANTES blocked infection of NP-2/CD4/CCR5 cells with HIV/SIV strains by 50–80%, as reported [37].

### Expression of FPRL1 mRNA in a wide variety of cells

We investigated the expression of FPRL1, CCR5, CXCR4, and GPR1 mRNA in cells originating from various types of human tissues by RT-PCR. CD4 and GAPDH mRNA were detected as controls. Fig. 2B shows that FPRL1 mRNA was detected abundantly in C8166 (T cell line), Molt4#8 (T cell line), U251MG (astrocytoma), and 293T (embryonal kidney) cells. Faint signals of FPRL1 mRNA were detected in HOS (osteosarcoma), U87/CD4 (CD4-transduced glioma cell line) cells, and PBLs. The expression levels of FPRL1 mRNA in Molt4#8, U251MG, and 293T cells were estimated to be comparable to those of CXCR4 in these cells. FPRL1 mRNA was, however, not detected in HepG2 (hepatoblastoma), Huh7 (hepatoma), or NP-2/CD4 (CD4-transduced glioma) cells. CCR5 mRNA was detected in Molt4#8, U251MG, and 293T cells, even though the levels were much lower than those of FPRL1 mRNA. CXCR4 mRNA was detected clearly in C8166 and Molt4#8 cells, and weakly in 293T, HepG2, HOS, Huh7, U251MG cells, and PBLs. CD4 mRNA was detected in BT-20N, C8166, Molt#3, NP-2/CD4, U87/CD4 cells, and PBLs. Similar amounts of GAPDH mRNA were detected in all cells tested here. No signal was seen in RNA samples without reverse transcriptase treatment, indicating that the chromosomal DNA did not contaminate the cDNA preparations. FPRL1 are expressed abundantly in various types of cells derived from not only lymphoid tissues, but also the brain.

## Discussion

The genomic diversity of HIV-1 is thought to be generated by the low fidelity of its reverse transcription and frequent recombination of the genome [39]. Mutation of amino acid sequences in the V3 domain of the Env can give HIV-1 the ability to use various GPCRs as coreceptors. Coreceptors other than CCR5 and CXCR4, that are related to the clinical involvement of the HIV-1 infection have not yet been clarified. We planned to identify novel coreceptors that can be frequently used not only by cell line-adapted HIV-1 strains, but also by primary HIV-1 isolates. In this study, we focused on a formylpeptide receptor, FPRL1, which is located genetically close to CKRs in the phylogenetic tree we made, containing three tyrosines in its NTR (Fig. 1 and see Additional file 1) as a candidate for a novel coreceptor.

First, we examined the coreceptor activity of FPRL1 for cell line-adapted HIV/SIV strains. We found that FPRL1 worked as a coreceptor for several HIV-1 strains, GUN-4V (coreceptor use: FPRL1-X4-GPR1) and GUN-7WT (FPRL1-R5-X4), but not GUN-1WT (R5-X4), GUN-1V (X4-GPR1), GUN-4WT (R5-X4), GUN-7V (X4-GPR1), IIIB (X4), Ba-L (R5), or SF162 (R5) (Fig. 3). Thus, FPRL1 use by cell line-adapted HIV-1 strains does not correlate with their use of CCR5, CXCR4 or GPR1. We showed that the R5-X4 phenotype of the GUN-4WT and GUN-7WT strains can be changed to the X4-GPR1 phenotype of GUN-4V and GUN-7V variants by one or two amino acid substitutions at the V3 loop [59]. Therefore, the V3 loop is thought to be a determinant of FPRL1 use, as well as GPR1 use, by HIV-1. Like other coreceptors for HIV-2 and SIV strains, FPRL1 was also efficiently used by R5-X4-dualtropic HIV-2 and SIV strains (Fig. 6).

Next, to examine the possibility that FPRL1 is involved in HIV-1 infection *in vivo*, we examined the susceptibility of NP-2/CD4/FPRL1 cells to primary HIV-1 isolates. It is generally thought that coreceptors other than CCR5 or CXCR4 are little used by primary HIV-1 isolates *in vivo *[40]. We found, however, that FPRL1 could work as a coreceptor for many primary HIV-1 isolates of subtype AE or C (Figs. 4 and 5). NP-2/CD4/FPRL1 cells were susceptible not only R5-X4 HIV-1 isolates (AG204, AG206, AG208, HCM305, and HCM308), but also R5 HIV-1 isolates (HCM342, and mSTD104) or an X4 HIV-1 isolate (HCM309) (see Additional file 2). NP-2/CD4/FPRL1 cells were not susceptible to R5-tropic HCM303 and X4-tropic mIDU101 isolates. Thus, we concluded that the FPRL1 use by HIV-1 strains does not coincide with the use of CCR5, CXCR4 or GPR1.

The ratio of FPRL1 use was as high as 80% for the primary HIV-1 isolates belonging to subtypes AE and C. This ratio for subtype B primary HIV-1 isolates was, however, much lower according to our preliminary examination (data not shown), suggesting that FPRL1 use may be linked to infection with subtypes AE and C HIV-1.

It is intriguing that only a few cell line-adapted HIV-1 strains could use FPRL1 as a coreceptor, whereas many primary HIV-1 isolates propagated in PBLs could do so. It is probable that a population of HIV-1 that uses FPRL1 as a coreceptor in addition to CCR5 or CXCR4 may have been lost in the course of propagation of HIV-1 strains *in vitro *using cell lines because it was markedly smaller than the HIV-1 population which did not use FPRL1, but did use CCR5 or CXCR4.

It has been shown that determinants for CCR5, CXCR4, and GPR1 uses by HIV-1 strains lie in the V3 domain of the Env protein [53,41]. Amino acid substitutions that are apparently linked to FPRL1 use could be identified in the V3 domain of GUN-1WT, GUN-4V, and GUN-7WT strains. That is, the amino acid substitutions at the tip of the V3 domain from proline to serine or from proline to threonine can affect FPRL1 use by HIV-1. Therefore, we examined the possibility that any specific amino acid substitution of the Env protein may give HIV-1 the ability to use FPRL1 as a coreceptor in addition to CCR5 or CXCR4 use. Primary isolates, AG204, AG206, AG208, HCM305, HCM308, HCM342, and mSTD104, were inoculated to, and propagated in, NP-2/CD4/FPRL1 cells in addition to NP-2/CD4/CCR5 or NP-2/CD4/CXCR4 cells. Then, HIV-1 DNA in these cells was subjected to DNA sequencing.

A few nucleotide substitutions were detected in the the V3 domain of the *env *gene between HIV-1 propagated in NP-2/CD4/FPRL1 cells and NP-2/CD4/CCR5 or NP-2/CD4/CXCR4 cells (see Additional file 3). The deduced amino acid sequences of the V3 domain of HIV-1 isolates propagating in NP-2/CD4/FPRL1 cells were identical to those propagated in NP-2/CD4/CCR5 or NP-2/CD4/CXCR4 cells. Furthermore, HIV-1 produced by NP-2/CD4/FPRL1 cells that had been infected with the HIV-1 isolates could infect NP-2/CD4/CCR5 or NP-2/CD4/CXCR4 cells (data not shown). Therefore, these primary isolates could use FPRL1 as a coreceptor in addition to CCR5 and/or CXCR4. It is possible that the determinants of FPRL1 use can not be separated from CCR5, CXCR4 or GPR1 use in HIV-1 strains. There is still another possibility that amino acid mutations in regions other than the V3 domain give HIV-1 strains the ability to use FPRL1 as a coreceptor.

fMLF peptides are bacterial products that have potent chemotactic activities for phagocytes. It was reported that FPRL1 activated by the fMLF peptide or peptides derived from the Env glycoprotein gp120 of HIV-1 interferes with the coreceptor function of CCR5 and CXCR4 by down-regulating them, and as a result, these peptides prevent HIV-1 infection [32,42,43]. Desensitization or down-regulation of CCR5 by the fMLF peptide has also been observed in human immature dendritic cells, on which both FPRL1 and CCR5 are expressed [42].

In this study, a partially inhibitory effect of the fMLF peptide on the FPRL1-mediated infection with HIV/SIV strains was observed, while anti-CD4 MoAb NuTH/I could almost completely block it (Figs. 7A and 7B). The regions of the coreceptors interacting with HIV or SIV are reported to be distributed in extracellular domains such as NTR and ECLs of GPCRs [44]. In contrast, the highly hydrophobic fMLF peptide has been reported to bind to the membrane-spanning region of FPRL1 [45]. Therefore, we assume that the difference in the binding site between the fMLF peptide and HIV-1 is one of the reasons why the fMLF peptide could not efficiently interfere with the interaction of FPRL1 with HIV-1.

The fMLF peptide also partially inhibited infection of NP-2/CD4/CCR5 cells with several HIV-2 or SIV strains (Fig. 7B). The amino acid sequences of the membrane-spanning regions are more conserved among GPCRs than those of NTRs and ECLs. Between FPRL1 and CCR5, the amino acid sequence homology of their membrane-spanning regions is about 30%, although that for their NTRs and ECLs is about 8.5% (data not shown). Therefore, the fMLF peptide might have a partially inhibitory effect on infection of NP-2/CD4/CCR5 cells with the CBL23 and mndGB-1 strains. The low, but apparent inhibitory effect of the fMLF peptide on HIV-2 infection mediated by CCR5 suggests that it can be a starting material to develop a novel anti-HIV drug.

Expression of the major coreceptor CCR5 or CXCR4 has been detected in various cells (Fig. 2A) [46]. Expression of FPRL1 mRNA was also detected in human cells derived from a wide variety of origins, such as PBLs, C8166 (T cells), Molt4#8 (T cells), 293T (embryonic kidney cells), HOS (osteosarcoma cells), U251MG (astrocytoma cell), and U87/CD4 (glioma cell) (Fig. 2A). In addition to these cells, expression of the FPRL1 gene has been reported to be detected in various types of cells such as neutrophils or phagocytes, and in many organs [31,46], even though their roles in human cells other than neutrophils remain to be elucidated. Roles of coreceptors other than CCR5 and CXCR4 in HIV-1 infection and the pathogenesis of AIDS should be investigated further.

## Conclusion

FPRL1 is a novel and efficient HIV/SIV coreceptor. In particular, it should be noted that FPRL1 is efficiently used by primary HIV-1 isolates. FPRL1 works as a bifunctional factor in HIV-1 infection. Namely, FPRL1 works not only as an inhibitory factor but also as an enhancing factor for HIV-1 to enter target cells. Expression of FPRL1 gene was detected in various types of tissues and cells. HIV1-infection mediated through FPRL1 may, therefore, play any important roles in progression of complicated symptoms of AIDS. The clinical significance of FPRL1 in HIV-1 infection *in vivo *should be further studied.

## Methods

### Cells

The human T cell line C8166 [48] and CCR5-transduced C8166 cells, C8166/CCR5 [49], were used to propagate viral stocks of HIV/SIV strains. NP-2/CD4 cells were established by transducing the CD4 gene into a human glioma cell line, NP-2, using a retrovirus vector as described elsewhere [16,49]. The human T cell lines C8166, C8166/CCR5, and Molt4#8 [50] were cultured in RPMI 1640 medium (NISSUI Co. Ltd., Tokyo, Japan) containing 10% fetal calf serum (FCS). The human osteosarcoma cell line HOS [51] and CD4-transduced human glioma cell line U87/CD4 [52,53], as well as NP-2/CD4 [49], NP-2/CD4/CCR5 [49], NP-2/CD4/CXCR4 [49], and NP-2/CD4/FPRL1 (see below) cells were cultured in Eagle's minimum essential medium (NISSUI Co., Inc., Tokyo, Japan) supplemented with 10% FCS. The human embryonal kidney cell line 293T [54], human hepatoblastoma cell line HepG2 [55], human hepatoma cell line Huh7, and human astrocytoma cell line U251MG [56] were maintained in Dulbecco's modified Eagle minimum essential medium (NISSUI Co., Inc., Tokyo, Japan) supplemented with 10% FCS. Brain-derived fibroblast-like BT-20/N cells [53,57], derived from the surgically dissected human brain tissue of a patient with glioma and thought to originate from brain blood vessels, were cultured in RPMI 1640 medium containing 10% FCS, endothelial cell growth supplements (BD Bioscience, Medford, MA) (10 μg/ml), and epidermal growth factor (10 ng/ml). Peripheral blood lymphocytes (PBLs) were isolated from healthy blood donors as previously described [58]. PBLs were stimulated with phytohemmagglutinin (PHA) and cultured in RPMI 1640 medium containing 10% FCS and recombinant IL-2 (100 U/ml).

### Virus strains

Cell line-adapted R5-X4 HIV-1 strains (GUN-1WT [57], GUN-4WT [59], and GUN-7WT [59]), GPR1-X4 HIV-1 variants (GUN-1V [57], GUN-4V [59], and GUN-7V [59]), an X4 HIV-1 strain (IIIB [60]), R5 HIV-1 strains (SF162 [61] and Ba-L [62]), HIV-2 strains (CBL23 [63], GH-1 [64], ROD/B [65], and SBL6669 [66]), and SIV strains (mac251 [67] and mndGB-1 [68]) were used. All of these HIV-1 strains are classified as subtype B based on their amino acid sequences of the Env protein [57-62]. The culture supernatants of C8166 cells infected with HIV/SIV strains except SF162, Ba-L, and mac251 strains, were harvested as viral stocks when cytopathicity was microscopically observed. SF162, Ba-L, and mac251 strains were propagated in C8166/CCR5 cells as previously described [49]. Primary HIV-1 isolates were propagated in PBLs and used in this study. Their origins, subtypes, and Genbank accession numbers of the *env *genes are described below. AG204 (Vietnum, subtype AE, Genbank accession number AB044003), AG206 (Vietnum, subtype AE, AB044005), AG208 (Vietnum, subtype AE, AB044007), HCM303 (Vietnum, subtype AE, AB044020), HCM305 (Vietnum, subtype AE, AB044022), HCM308 (Vietnum, subtype AE, AB044024), HCM309 (Vietnum, subtype AE, AB044025), and HCM342 (Vietnum, subtype AE, AB044034), mIDU101 (Myanmar, subtype C, AB097871), mSTD104 (Myanmar, subtype C, unpublished).

### PCR primers

Oligonucleotide primers were synthesized (Proligo K. K., Tokyo, Japan) to detect the expression of mRNA for CD4, glyceraldehyde-3-phosphate dehydrogenase (GAPDH), CCR5, CXCR4, GPR1, or FPRL1 by reverse transcription (RT)-PCR. Each gene name, followed by each sense and antisense primer name, their nucleotide sequences and positions in the open reading frame [DDBJ/EMBL/Genbank accession number] are described below. CD4: CD4CN, 5'-ATGAACCGGGGAGTCCCTTTTAGGCACTTG-3' (sense: from the 1st to the 30th position, i. e. 1st–30th); and CD4CR, 5'-TCAAATGGGGCTACATGTCTTCTGAAACCG-3' (anti-sense: 1,039th–1,068th) [DDBJ/EMBL/Genbank accession number, NM000616]. GAPDH: G3PDHN, 5'-TGAAGGGTCGGAGTCAACGGATTTGGT-3' (sense, 11th–36th); and GAPDHR, 5'-TAGACGGCAGGTCAGGTCCACCAC-3' (antisense, 724th–747th) [BT006893]. PCR primers used to detect GPCR cDNA are as follows: CCR5: CCR5CN, 5'-ATGGATTATCAAGTGTCAAGTCCAATCTAT-3' (sense,1st–30th); and CCR5CR, 5'-TCACAAGCCCACAGATATTTCCTGCTCCCC-3' (antisense, 1,001st–1,030th) [NM000579]. CXCR4: CXCR4CN, 5'-ATGGAGGGGATCAGTATATACACTTCAGAT-3' (sense: 1st–30th); and CXCR4CR, 5'-'TTAGCTGGAGTGAAAACTTGAAGACTCAGA-3' (antisense, 979–1,008th) [NM003467]. FPRL1: FPRL1CN, 5'-ATGGAAACCAACTTCTCCACTCCTCTGAAT-3' (sense, 1st–30th); and FPRL1CR, 5'-TCACATTGCCTGTAACTCAGTCTCTGCAGG-3' (antisense, 1,027rd–1,056nd) [M76672]. GPR1: GPR1CN, 5'-ATGGAAGATTTGGAGGAAACATTATTTGAA-3' (sense, 1st–30th); and GPR1CR, 5'-TTATTGAGCTGTTTCCAGGAGACACAGATT-3' (antisense, 1,039th–1,068th) [U13666].

### Detection of GPCR mRNA

Total RNA was isolated from human cells using an RNA extraction kit, SepaGene (Sanko-Junyaku Inc., Tokyo, Japan), in accordance with the manufacturer's protocol. cDNA for the total cellular RNA was constructed as described elsewhere [49]. mRNA expression for CCR5, CD4, CXCR4, GPR1, FPRL1, and GAPDH was detected by PCR of cDNA preparations using the sense and antisense primer pairs described above [49]. Amplified cDNA was detected by 1% (w/v) agarose gel electrophoresis.

### Cloning of the FPRL1 gene

A DNA fragment encoding the entire open reading frame (ORF) of the FPRL1 gene was amplified by RT-PCR using the FPRL1-specific primers, FPRL1CN and FPRL1CR, and cDNA constructed from the total RNA isolated from C8166 cells. The ORF DNA of the FPRL1 gene was cloned into the TA-cloning plasmid pDrive (QIAGEN K. K., Tokyo, Japan) and the plasmid obtained was designated pDrive/FPRL1. The DNA fragment containing FPRL1 ORF was isolated from the pDrive/FPRL1 plasmid by *Eco*RI digestion and subcloned into the expression plasmid pCX-bsr [69]. The FPRL1 plasmid obtained was designated pCX-puro/FPRL1. The cloned FPRL1 gene was sequenced and found to be 100% homologous in terms of amino acid sequences to the reported gene [M76672] [30].

### Establishment of FPRL1-expressing cells

An FPRL1-expressing cell line was established as follows. The plasmid harboring the receptor gene for ecotropic murine leukemia virus (MuLV) and hygromycin-resistance gene was transfected into NP-2/CD4 cells and hygromycin-resistant cells were selected as reported previously [16]. BOSC23 cells [70] were transfected with the pCX-bsr/FPRL1 plasmid and ecotropic MuLV pseudotype was produced from the cells. NP-2/CD4 cells were infected with the ecotropic MuLV pseudotype produced by BOSC23 cells. The blasticidin-resistant NP-2/CD4 cells were selected through cultivation in medium containing blasticidin (10 μg/ml) (CALBIOCHEM, San Diego, CA) for two weeks. Surviving cells were designated NP-2/CD4/FPRL1. NP-2/CD4/CCR5, NP-2/CD4/CXCR4, and NP-2/CD4/GPR1 cells were established previously [16,23]. The expressions of mRNAs for CCR5, CXCR4, GPR1, and FPRL1 genes in these cells were detected by RT-PCR using cDNA and the PCR primers prepared as described above.

### Infection assay

NP-2/CD4, NP-2/CD4/CCR5, NP-2/CD4/CXCR4, NP-2/CD4/FPRL1 and NP-2/CD4/GPR1 cells (5 × 10^4^) were seeded into 24-well culture plates 24 h prior to viral inoculation. These cells were exposed to HIV/SIV in an amount of virus corresponding to 1 × 10^4 ^cpm of the reverse transcriptase activity as previously described [71]. After incubation for two hours, the cells were washed three times with E-MEM containing 10% FCS and then cultured in 500 μl of fresh medium at 37°C. The cells were passaged every two days.

### Determination of the effects of an anti-CD4 monoclonal antibody, GPCR ligands, and tyrosine sulfation on HIV-1 infection

To determine CD4 dependency of HIV infection, NP-2/CD4/CCR5 and NP-2/CD4/FPRL1 cells were cultured in E-MEM containing a serially diluted anti-CD4 monoclonal antibody (MoAb), Nu-TH/I (Nichirei Inc., Tokyo, Japan), at 37°C for two hours. The cells were incubated in E-MEM with or without Nu-TH/I MoAb at 37°C for two hours and then exposed to HIV-1 in an amount corresponding to 1 × 10^4 ^cpm of RT activity. After removing the inocula, the cells were incubated at 37°C in E-MEM containing 10% (v/v) FCS for four days.

To investigate the effects of ligands on infection of cells with HIV/SIV strains, NP-2/CD4/CCR5 or NP-2/CD4/FPRL1 cells were incubated in E-MEM containing RANTES (100 μg/ml) (BIOCARTA US, San Diego, CA) or forymyl-Met-Leu-Phe (fMLF) peptide (100 μg/ml) (WAKO Jun-yaku, Inc., Tokyo, Japan) at 37°C for three hours. Then, the cells were exposed to HIV-1 as described above.

CCR5 and FPRL1 contain several tyrosine residues in their NTRs and ECLs, but only NTRs harbor the signal sequence for tyrosine sulfation [73]. To clarify the effect of tyrosine sulfation in NTR of CCR5 and FPRL1, NP-2/CD4/CCR5 and NP-2/CD4/FPRL1 cells were incubated in E-MEM containing an inhibitor of tyrosine sulfation, NaClO3 (10 mM), for 48 hours and then inoculated with viruses.

### Detection of HIV-/SIV-infected cells

The susceptibilities of the cells described above to HIV/SIV were determined by indirect immunofluorescence assay (IFA), which detects HIV-1-, HIV-2-, or SIV-antigens expressed in infected cells, as previously reported [71]. A pool of sera derived from HIV-1-infected humans or SIVmac-infected macaques was used as the first antibody [16,49]. Infection was checked on days 2, 4, and 6 after inoculation.

### Phylogenetic analysis

The multiple alignment of the amino acid sequences of 20 CKRs and 16 GPCRs closely related to CKRs and their phylogenetic tree was constructed using the ClustalW program [72]. GPCR names and their abbreviations in the protein database SWISS PROT were as follows: type-1 angiotensin II receptor AG2R [DDBJ/EMBL/Genbank accession number, M91464], type-2 angitensin II receptor AG22 [U20860], type-1B angiotensin II receotor AG2S [D13814], apelin receptor APJ [U03642], B1 bradykinin B1 receptor BRB1 [U12512], B2 bradykinin receptor BRB2 [M88714], C3a anaphylatoxin receptor C3AR [U28488], C5a anaphylatoxin receptor C5AR [X58674], C5a anaphylatoxin receptor C5L2 [B038237], CC-CKR CCR1 [NM001295], CC-CKR CCR2b [NM000648], CC-CKR CCR3 [NM001837], CC-CKR CCR4 [NM005508], CC-CKR CCR5 [NM000579], CC-CKR CCR6 [NM004367], CC-CKR CCR7 [NM001838], CC-CKR CCR8 [NM005201], CC-CKR CCR9 [NM006641], CC-CKR CCR10 [NM016602], CC-CKR CCR11 [AF110640], CX3C-CKR CX3CR1 [NM001337], CXC-CKR CXCR1 [NM000634], CXC-CKR CXCR2 [NM001557], CXC-CKR CXCR3 [NM001504], CXC-CKR CXCR4 [NM003467], CXC-CKR CXCR5 [NM001716], CXC-CKR CXCR6 [NM006564], CC-CKR D6 [NM001296], formylpeptide receptor 1 FPRL1 [M76672], formylpeptide receptor 2 [M37128], FPRL1-related receptor FPR1 [M76673], proteinase-activated receptor 1 PAR1 [M62424], protease-activated receptor 2 PAR2 [Z49993], protease-activated receptor 3 PAR3 [U92971], and protease-activated receptor 4 PAR4 [AF080214].

## Authors' contributions

NS conceived and designed this study, NS also carried out the molecular genetic and virological studies, and drafted the manuscript, AT carried out establishment of cell lines, AO and TM carried out biochemical studies. AH, CA, SK, TO, and YT participated in virus preparation and their characterization, HH played important roles in coordination of this study and helped to draft the manuscript.

## Supplementary Material

Additional file 1 Table 1. HIV/SIV coreceptors and formylpeptide receptors, and amino acid sequences of their NTRs. Click here for file

Additional file 2 Table 2. Use of GPCRs as coreceptors by HIV-1, HIV-2, and SIV. Click here for file

Additional file 3 Table 3. FPRL1 use and amino acid sequences of the V3 domain of HIV-1 strains. Click here for file
