# Supplementary material for: A formylpeptide receptor, FPRL1, acts as an efficient coreceptor for primary isolates of human immunodeficiency virus
Source: Retrovirology. 2008 Jun 25;5:52. doi: 10.1186/1742-4690-5-52 (PMC2453146; doi:10.1186/1742-4690-5-52)
Supplement: Additional file 1 — Table 1. HIV/SIV coreceptors and formylpeptide receptors, and amino acid sequences of their NTRs. [file 1742-4690-5-52-S1.ppt]

## Slide 1
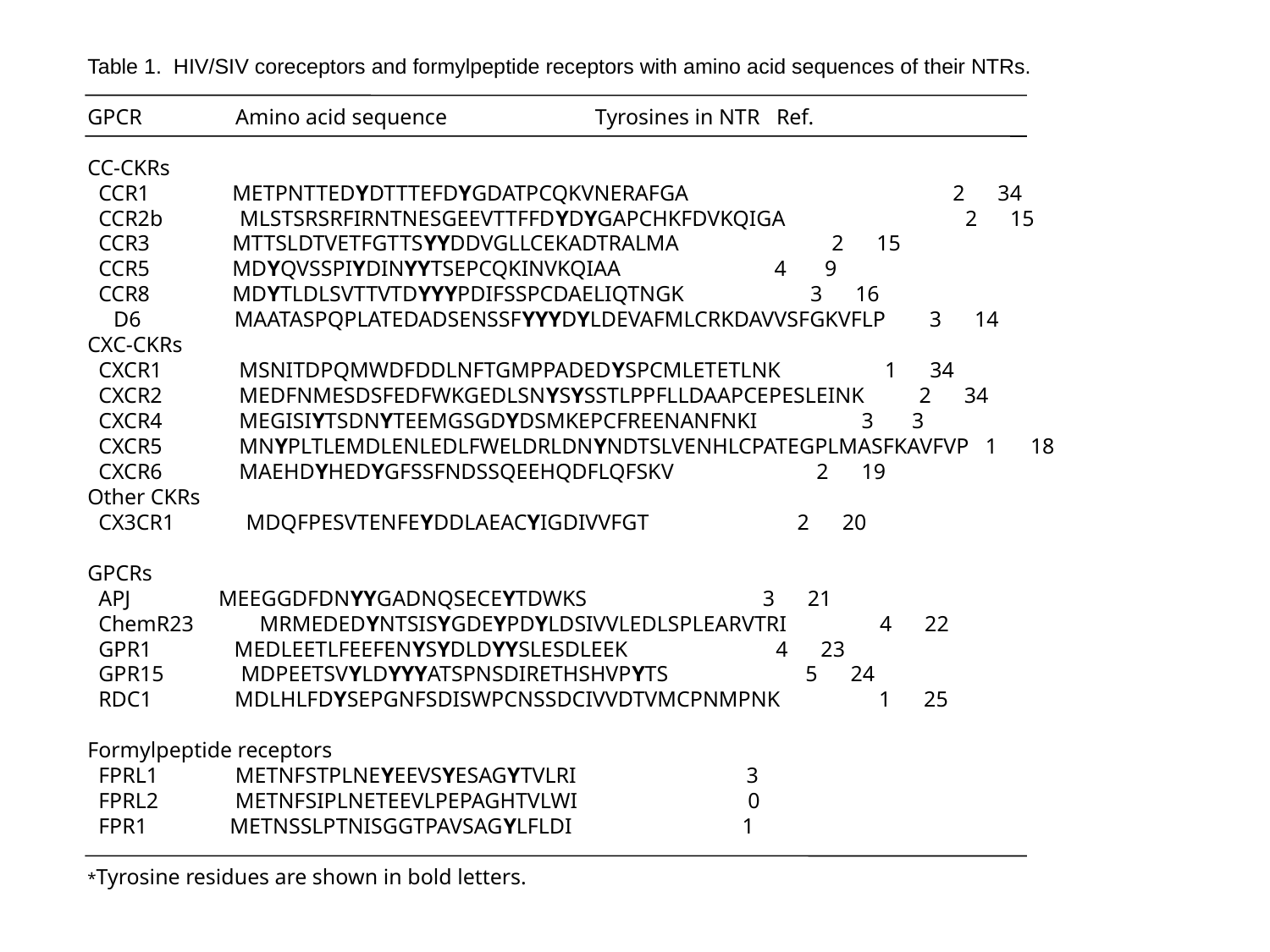

Table 1. HIV/SIV coreceptors and formylpeptide receptors with amino acid sequences of their NTRs.
GPCR Amino acid sequence Tyrosines in NTR Ref.
CC-CKRs
 CCR1 METPNTTEDYDTTTEFDYGDATPCQKVNERAFGA　　　　　　　　　　　　2 34
 CCR2b MLSTSRSRFIRNTNESGEEVTTFFDYDYGAPCHKFDVKQIGA　　　　　　　　2 15
 CCR3 MTTSLDTVETFGTTSYYDDVGLLCEKADTRALMA 　2 15
 CCR5 MDYQVSSPIYDINYYTSEPCQKINVKQIAA 4 9
 CCR8 MDYTLDLSVTTVTDYYYPDIFSSPCDAELIQTNGK 3 16
　D6 MAATASPQPLATEDADSENSSFYYYDYLDEVAFMLCRKDAVVSFGKVFLP 3 14
CXC-CKRs
 CXCR1 MSNITDPQMWDFDDLNFTGMPPADEDYSPCMLETETLNK 1 34
 CXCR2 MEDFNMESDSFEDFWKGEDLSNYSYSSTLPPFLLDAAPCEPESLEINK 2 34
 CXCR4 MEGISIYTSDNYTEEMGSGDYDSMKEPCFREENANFNKI 3 3
 CXCR5 MNYPLTLEMDLENLEDLFWELDRLDNYNDTSLVENHLCPATEGPLMASFKAVFVP 1 18
 CXCR6 MAEHDYHEDYGFSSFNDSSQEEHQDFLQFSKV 2 19
Other CKRs
 CX3CR1 MDQFPESVTENFEYDDLAEACYIGDIVVFGT 2 20
GPCRs
 APJ MEEGGDFDNYYGADNQSECEYTDWKS 3 21
 ChemR23 MRMEDEDYNTSISYGDEYPDYLDSIVVLEDLSPLEARVTRI 4 22
 GPR1 MEDLEETLFEEFENYSYDLDYYSLESDLEEK 4 23
 GPR15 MDPEETSVYLDYYYATSPNSDIRETHSHVPYTS 5 24
 RDC1 MDLHLFDYSEPGNFSDISWPCNSSDCIVVDTVMCPNMPNK 1 25
Formylpeptide receptors
 FPRL1 METNFSTPLNEYEEVSYESAGYTVLRI 3
 FPRL2 METNFSIPLNETEEVLPEPAGHTVLWI 0
 FPR1 METNSSLPTNISGGTPAVSAGYLFLDI 1
*Tyrosine residues are shown in bold letters.
