# Supplementary figures and images for: A formylpeptide receptor, FPRL1, acts as an efficient coreceptor for primary isolates of human immunodeficiency virus
Source: Retrovirology. 2008 Jun 25;5:52. doi: 10.1186/1742-4690-5-52 (PMC2453146; doi:10.1186/1742-4690-5-52)

## Slide 1
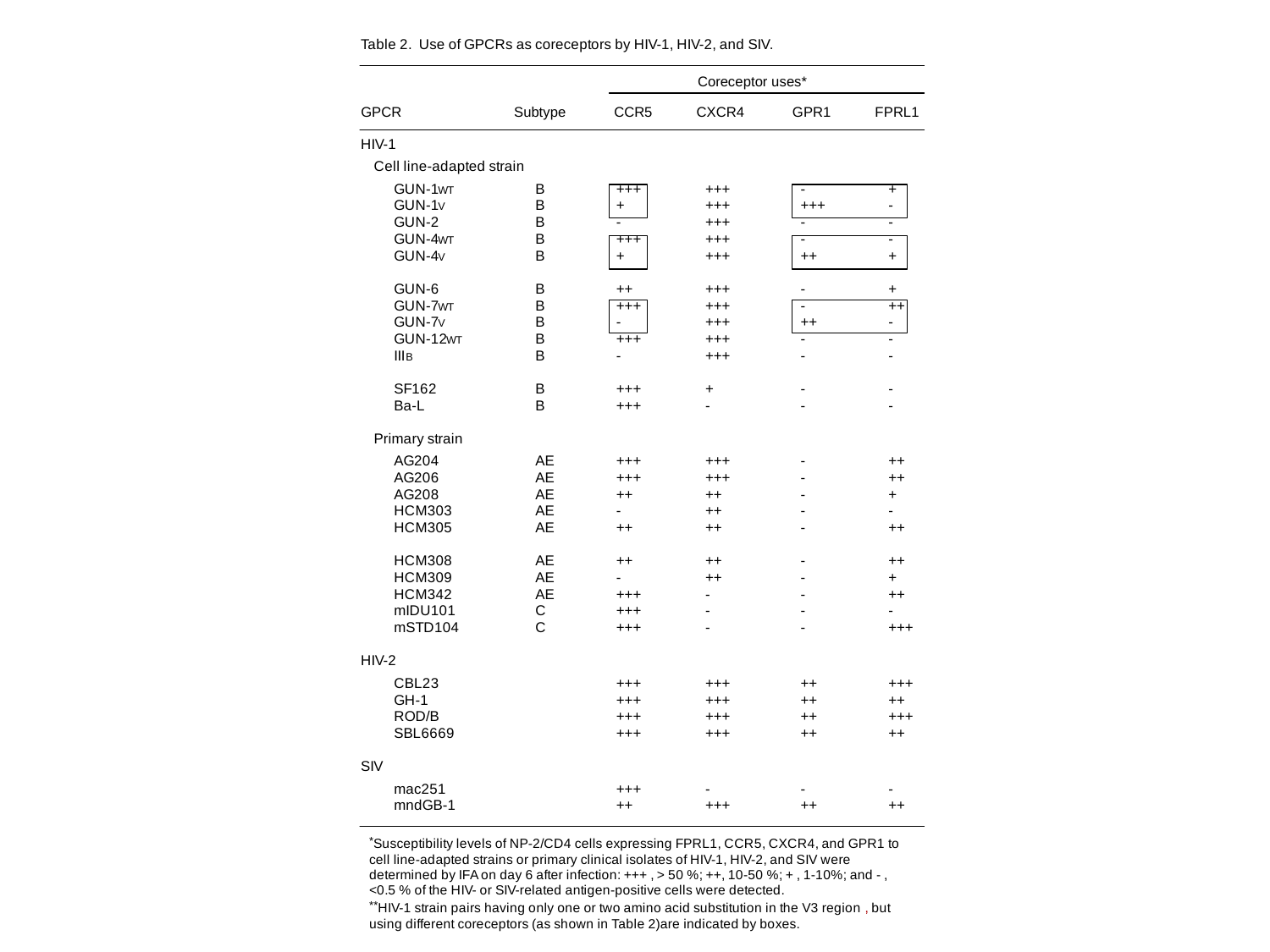

Coreceptor uses*
GPCR Subtype CCR5 CXCR4 GPR1 FPRL1

Supplement: Additional file 2 — Table 2. Use of GPCRs as coreceptors by HIV-1, HIV-2, and SIV. [file 1742-4690-5-52-S2.ppt]
