# Supplementary material for: A formylpeptide receptor, FPRL1, acts as an efficient coreceptor for primary isolates of human immunodeficiency virus
Source: Retrovirology. 2008 Jun 25;5:52. doi: 10.1186/1742-4690-5-52 (PMC2453146; doi:10.1186/1742-4690-5-52)
Supplement: Additional file 3 — Table 3. FPRL1 use and amino acid sequences of the V3 domain of HIV-1 strains. [file 1742-4690-5-52-S3.ppt]

## Slide 1
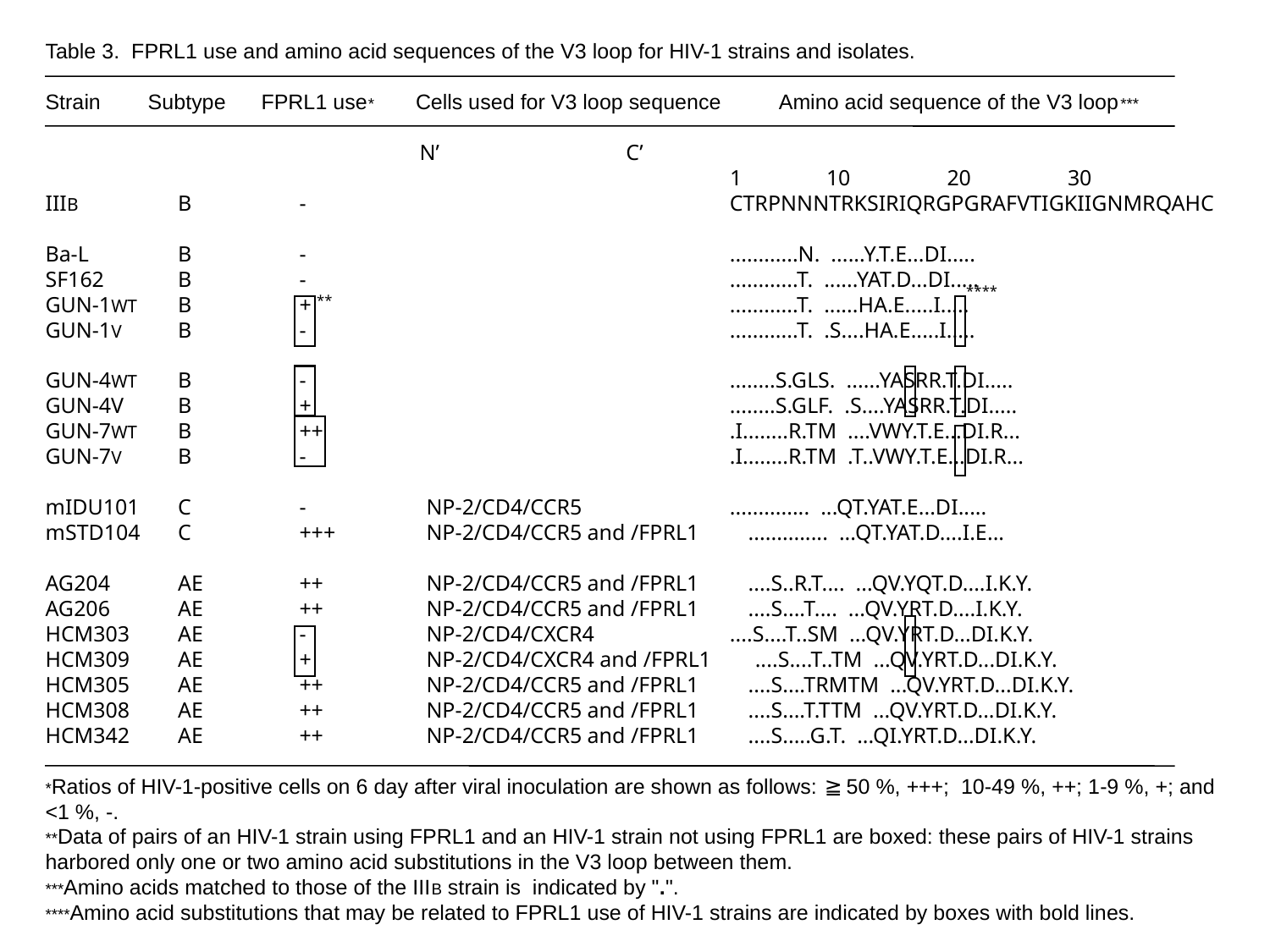

Table 3. FPRL1 use and amino acid sequences of the V3 loop for HIV-1 strains and isolates.
Strain Subtype FPRL1 use* Cells used for V3 loop sequence Amino acid sequence of the V3 loop***
 N’ C’
					 100000001000000000200000000030
IIIB	 B	-			 CTRPNNNTRKSIRIQRGPGRAFVTIGKIIGNMRQAHC
Ba-L	 B	-			 ............N. ......Y.T.E...DI.....
SF162	 B	-			 ............T. ......YAT.D...DI.....
GUN-1WT	 B	+			 ............T. ......HA.E.....I.....
GUN-1V	 B	-			 ............T. .S....HA.E.....I.....
GUN-4WT	 B	-			 ........S.GLS. ......YASRR.T.DI.....
GUN-4V	 B	+			 ........S.GLF. .S....YASRR.T.DI.....
GUN-7WT	 B	++			 .I........R.TM ....VWY.T.E...DI.R...
GUN-7V	 B	-			 .I........R.TM .T..VWY.T.E...DI.R...
mIDU101	 C	-	NP-2/CD4/CCR5	 .............. ...QT.YAT.E...DI.....
mSTD104	 C	+++	NP-2/CD4/CCR5 and /FPRL1 .............. ...QT.YAT.D....I.E...
AG204	 AE	++	NP-2/CD4/CCR5 and /FPRL1 ....S..R.T.... ...QV.YQT.D....I.K.Y.
AG206	 AE	++	NP-2/CD4/CCR5 and /FPRL1 ....S....T.... ...QV.YRT.D....I.K.Y.
HCM303	 AE	-	NP-2/CD4/CXCR4	 ....S....T..SM ...QV.YRT.D...DI.K.Y.
HCM309	 AE	+	NP-2/CD4/CXCR4 and /FPRL1 ....S....T..TM ...QV.YRT.D...DI.K.Y.
HCM305	 AE	++	NP-2/CD4/CCR5 and /FPRL1 ....S....TRMTM ...QV.YRT.D...DI.K.Y.
HCM308	 AE	++	NP-2/CD4/CCR5 and /FPRL1 ....S....T.TTM ...QV.YRT.D...DI.K.Y.
HCM342	 AE	++	NP-2/CD4/CCR5 and /FPRL1 ....S.....G.T. ...QI.YRT.D...DI.K.Y.
*Ratios of HIV-1-positive cells on 6 day after viral inoculation are shown as follows: ≧ 50 %, +++; 10-49 %, ++; 1-9 %, +; and
<1 %, -.
**Data of pairs of an HIV-1 strain using FPRL1 and an HIV-1 strain not using FPRL1 are boxed: these pairs of HIV-1 strains
harbored only one or two amino acid substitutions in the V3 loop between them.
***Amino acids matched to those of the IIIB strain is indicated by ".".
****Amino acid substitutions that may be related to FPRL1 use of HIV-1 strains are indicated by boxes with bold lines.
****
**
